# Combining Partial Specifications using Alternating Interface Automata

**DOI:** 10.1007/978-3-030-45234-6_23

**Published:** 2020-03-13

**Authors:** Ramon Janssen

**Affiliations:** 8 University of Paderborn, Paderborn, Germany; 9 ICREA, Open University of Catalonia, Barcelona, Spain; Radboud University, Nijmegen, The Netherlands

## Abstract

To model real-world software systems, modelling paradigms should support a form of compositionality. In interface theory and model-based testing with inputs and outputs, *conjunctive* operators have been introduced: the behaviour allowed by composed specification $s_1 \wedge s_2$ is the behaviour allowed by both partial models $s_1$ and $s_2$. The models at hand are non-deterministic *interface automata*, but the interaction between non-determinism and conjunction is not yet well understood. On the other hand, in the theory of *alternating automata*, conjunction and non-determinism are core aspects. Alternating automata have not been considered in the context of inputs and outputs, making them less suitable for modelling software interfaces. In this paper, we combine the two modelling paradigms to define *alternating interface automata* (AIA). We equip these automata with an observational, trace-based semantics, and define testers, to establish correctness of black-box interfaces with respect to an AIA specification.

## 1 Introduction

The challenge of software verification is to ensure that software systems are correct, using techniques such as model checking and model-based testing. To use these techniques, we assume that we have an abstract specification of a system, which serves as a description of what the system should do. A popular approach is to model a specification as an automaton. However, the huge number of states in typical real-world software systems quickly makes modelling with explicit automata infeasible. A form of compositionality is therefore usually required for scalability, so that a specification can be decomposed into smaller and understandable parts. Parallel composition is based on a structural decomposition of the modelled system into components, and it thus relies on the assumption that components themselves are small and simple enough to be modelled. This assumption is not required for logical composition, in which partial specification models of the same component or system are combined in the manner of logical conjunction. Formally, for a composition to be conjunctive, the behaviour allowed by $s_1 \wedge s_2$ is the behaviour allowed by both partial specifications $s_1$ and

* Funded by the Netherlands Organisation of Scientific Research (NWO-TTW), project 13859: SUMBAT - SUpersizing Model-BAsed Testing

$s_2$. Such a composition is important for scalability of modelling, as it allows writing independent partial specifications, sometimes called view modelling [3]. On a fundamental level, specifications can be seen as logical statements about software, and the existence of conjunction on such statements is only natural. Conjunctive operators have been defined in many language-theoretic modelling frameworks, such as for regular expressions [12] and process algebras [5].

### 1.1 Conjunction for Inputs and Outputs

A conjunctive operator $\wedge$ has also been introduced in many automata frameworks for formal verification and testing, such as interface theory [8], ioco theory [3] and the theory of substitutivity refinement [7]. Within these theories, systems are modelled as *labelled transition systems* [15] or *interface automata* [1] (IA), and actions are divided into inputs and outputs.

An informal example of some (partial) specification models, as could be expressed in these theories, is shown by the automata in Figure 1, in which inputs are labelled with question marks, and outputs with exclamation marks. The specifications represent a vending machine with two input buttons (?a and ?b), which provides coffee (!c) and tea (!t) as outputs, optionally with milk (!c+m and !t+m). The first model, $p$, specifies that after pressing button ?a, the machine dispenses coffee. The second model, $q$, specifies that after pressing button ?b, the machine has a choice between dispensing tea, or tea with milk. The third model, $r$, is similar, but uses non-determinism to specify that button ?b results in coffee with milk or tea with milk.

The fourth model, $p \wedge q \wedge r$, states that all former three partial models should hold. Here, we use the definition of $\wedge$ from [3], but the definition from [7] is similar. An input is specified in the combined model if it is specified in any partial model, making both buttons ?a and ?b specified. Additionally, an output is allowed in the combined model if it is allowed by all partial models, meaning that after button ?b, only tea with milk is allowed.

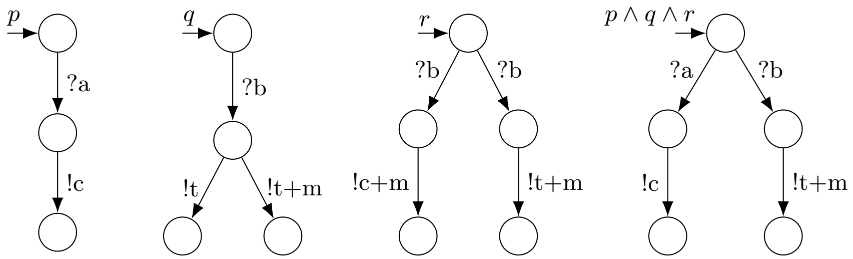

**Fig. 1.** Three independent specifications for a vending machine, and their conjunction.

### 1.2 Conjunctions of states

This form of conjunctive composition acts as an operator on entire models. However, a partial specification could also describe the expected behaviour of a particular state of the system, other than the initial state. For example, suppose that the input ?on turns the vending machine on, after which the machine should behave as specified by $p$, $q$ and $r$ from Figure 1. This, by itself, is also a specification, illustrated by $s$ in Figure 2. However, the formal meaning of this model is unclear: transitions connect states, whereas $p \wedge q \wedge r$ is not a state but an entire automaton. A less trivial case is partial specification $t$, also in Figure 2: after obtaining any drink by input ?take, we should move to a state where we can obtain a drink as described by specifications $p$, $q$, $r$ and $t$. Thus, we combine conjunctions with a form of recursion. This cannot easily be formalized using $\wedge$ as an operator on automata, like in [3,7,8]. Defining conjunction as a composition on individual states would provide a formal basis for these informal examples.

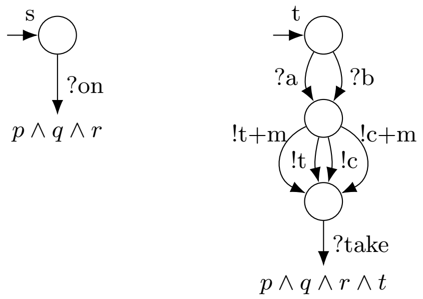

**Fig. 2.** Two specifications with transitions to a conjunction.

Conjunctions of states are a main ingredient of *alternating automata* [6], in which conjunctions and non-determinism alternate. Here, non-determism acts as logical disjunction, dually to conjunction. Because of this duality, both conjunction and disjunction are treated analogously: both are encoded in the transition relation of the automaton. This contrasts the approach of defining conjunction directly on IAs, where non-determinism is encoded in the transition relation of the IA, whereas conjunction is added as an operator on IAs, leaving the duality between the two unexploited. In fact, the conjunction-operator in [3] even requires that any non-determinism in its operands is removed first, by performing an exponential determinization step. For example, model $r$ in Figure 1 is non-deterministic, and must be determinized to the form of model $q$ before $p \wedge q \wedge r$ is computed. This indicates that it is hard to combine conjunction and non-determinism in an elegant way, without understanding their interaction.

Despite their inherent support for conjunction, alternating automata are not entirely suitable for modeling the behaviour of software systems, since they lack the distinction between inputs and outputs. In this respect, alternating automata are similar to deterministic finite automata (DFAs). Distinguishing inputs and outputs in an IA allows modelling of software systems in a less abstract way than with the homogeneous alphabet of actions of DFAs and alternating automata.

### 1.3 Contributions

We combine concepts from the worlds of interface theory and alternating automata, leading to Alternating Interface Automata (AIAs), and show how these can be used in the setting of a trace semantics for observable inputs and outputs. We provide a solid formal basis of AIAs, by

- combining alternation with inputs and outputs (Section 3.1),
- defining a trace semantics for AIAs (Section 3.2), by lifting the *input-failure refinement* semantics for non-deterministic interface automata [11] to AIAs,
- providing insight into the semantics of an AIA, by defining a determinization operator (Section 3.3) and a transformation between IAs and AIAs (Section 3.4), and
- defining testers (Section 4), which represent practical testing scenarios for establishing input-failure refinement between a black-box implementation IA and a specification AIA, analogously to ioco test case generation [15].

The definition of input-failure refinement [11] is based upon the observation that, for a non-deterministically reached set of states $Q$, the observable outputs of that set are the union of the outputs of the individual states in $Q$, whereas the specified inputs for $Q$ are the intersection of the inputs specified in individual states in $Q$. For conjunction, we invert this: outputs allowed by a conjunction of states are captured by the intersection, whereas specified inputs are captured by the union. In this way, our AIAs seamlessly combine the duality between conjunction and non-determinism with the duality between inputs and outputs.

Proofs can be found in the extended technical report [10].

## 2 Preliminaries

We first recall the definition of interface automata [1] and input-failure refinement [11]. The original definition of IAs [1] allows at most one initial state, but we generalize this to sets of states. Moreover, [1] supports internal actions, which we do not need. Transitions are commonly encoded by a relation, whereas we use a function.

**Definition 1.** *An Interface Automaton (IA) is a 5-tuple* $(Q, I, O, T, Q^0)$*, where*

- $Q$ *is a set of states,*
- $I$ *and* $O$ *are disjoint sets of input and output actions, respectively,*
- $T : Q \times (I \cup O\}) \rightarrow \mathcal{P}(Q)$ *is an image-finite transition function (meaning that* $T(q, \ell)$ *is finite for all* $q$ *and* $\ell$*), and*
- $Q^0 \subseteq Q$ *is a finite set of initial states.*

*The domain of IAs is denoted* $\mathcal{IA}$*. For* $s \in \mathcal{IA}$*, we refer to its respective elements by* $Q_s$*,* $I_s$*,* $O_s$*,* $T_s$*,* $Q^0_s$*. For* $s_1, s_2, \ldots, s_A, s_B, \ldots$ *a family of IAs, we write* $Q_j$*,* $I_j$*,* $O_j$*,* $T_j$ *and* $Q^0_j$ *to refer to the respective elements, for* $j = 1, 2, \ldots, A, B, \ldots$*.*

In examples, we represent IAs graphically as in Figure 1. For the remainder of this paper, we assume fixed input and output alphabets $I$ and $O$ for IAs, with $L = I \cup O$. For (sets of) sequences of actions, $*$ denotes the Kleene star, and $\epsilon$ denotes the empty sequence. We define auxiliary notation in the style of [15].

**Definition 2.** *Let $s \in \mathcal{IA}$, $Q \subseteq Q_s$, $q, q' \in Q_s$, $\ell \in L$ and $\sigma \in L^*$. We define*

$$
\begin{aligned}
q \xrightarrow{\epsilon}_s q' &\Leftrightarrow q = q' & q \xrightarrow{\sigma\ell}_s q' &\Leftrightarrow \exists r \in Q_s : q \xrightarrow{\sigma}_s r \wedge q' \in T_s(r, \ell)\\
q \xrightarrow{\sigma}_s &\Leftrightarrow \exists r \in Q_s : q \xrightarrow{\sigma}_s r & q \not\xrightarrow{\sigma}_s &\Leftrightarrow \neg(q \xrightarrow{\sigma}_s)\\
\text{traces}_s(q) &= \{\sigma \in L^* \mid q \xrightarrow{\sigma}_s\} & Q \text{ after}_s\, \sigma &= \{r \in Q_s \mid \exists r' \in Q : r' \xrightarrow{\sigma}_s r\}\\
\text{traces}(s) &= \bigcup_{q \in Q_s^0} \text{traces}_s(q) & s \text{ after } \sigma &= Q_s^0 \text{ after}_s\, \sigma
\end{aligned}
$$

$$
\text{out}_s(Q) = \{x \in O \mid \exists q \in Q : q \xrightarrow{x}_s\} \quad \text{in}_s(Q) = \{a \in I \mid \forall q \in Q : q \xrightarrow{a}_s\}
$$

$$
\begin{aligned}
q \textit{ is a } \text{sink-state } \textit{of } s &\iff \forall \ell \in L : T_s(q, \ell) \subseteq \{q\}\\
s \textit{ is } \text{input-enabled} &\iff \forall q \in Q_s : \text{in}_s(q) = I\\
s \textit{ is } \text{deterministic} &\iff \forall \sigma \in L^*, |s \text{ after } \sigma| \leq 1
\end{aligned}
$$

We omit the subscript for interface automaton $s$ when clear from the context.

We use IAs to represent black-box systems, which can produce outputs, and consume or refuse inputs from the environment. This entails a notion of observable behaviour, which we define in terms of *input-failure traces* [11].

**Definition 3.** *For any input action $a$, we denote the* input-failure of *$a$ as $\overline{a}$. Likewise, for any set of inputs $A$, we define $\overline{A} = \{\overline{a} \mid a \in A\}$. The domain of* input-failure traces *is defined as $\mathcal{FT}_{I,O} = L^* \cup L^* \cdot \overline{I}$. For $s \in \mathcal{IA}$, we define*

$$
\text{Ftraces}(s) = \text{traces}(s) \cup \{\sigma\overline{a} \mid \sigma \in L^*, a \in I, a \notin \text{in}(s \text{ after } \sigma)\}
$$

Thus, a trace $\sigma\overline{a}$ indicates that $\sigma$ leads to a state where $a$ is not accepted, e.g. a greyed-out button which cannot be clicked.

Any such set of input-failure traces is prefix-closed. Input-failure traces are the basis of *input-failure refinement*, which we will now explain briefly. This refinement relation was introduced in [11] to bridge the gap between alternating refinements [1,2] and ioco theory [15]. Similarly to normal trace inclusion, the idea is that an implementation may only show a trace if a specification also shows this trace. Moreover, the most permissive treatment of an input is to fail it, so if a specification allows an input failure, then it also must allow acceptance of that input, as expressed by the *input-failure closure*.

**Definition 4.** *Set $S \subseteq \mathcal{FT}_{I,O}$ of input-failure traces is* input-failure closed *if, for all $\sigma \in L^*$, $a \in I$ and $\rho \in \mathcal{FT}_{I,O}$, $\sigma\overline{a} \in S \implies \sigma a\rho \in S$. The* input-failure closure *of $S$ is the smallest input-failure closed superset of $S$, that is,* $\text{fcl}(S) = S \cup \{\sigma a \rho \mid \sigma\overline{a} \in S, \rho \in \mathcal{FT}_{I,O}\}$.

Input-failure refinement *and* input-failure equivalence *on IAs are respectively defined as*

$$s_1 \leq_{if} s_2 \iff \text{Ftraces}(s_1) \subseteq \text{fcl}(\text{Ftraces}(s_2)), \textit{ and}$$
$$s_1 \equiv_{if} s_2 \iff s_1 \leq_{if} s_2 \wedge s_2 \leq_{if} s_1.$$

The input-failure closure of the Ftraces serves as a canonical representation of the behaviour of an IA. That is, two models are input-failure equivalent if and only if the closure of their input-failure traces is the same, as stated in Proposition 5.

**Proposition 5.** *[11] Let $s_1, s_2 \in \mathcal{IA}$. Then*

$$s_1 \leq_{if} s_2 \iff \text{fcl}(\text{Ftraces}(s_1)) \subseteq \text{fcl}(\text{Ftraces}(s_2))$$
$$s_1 \equiv_{if} s_2 \iff \text{fcl}(\text{Ftraces}(s_1)) = \text{fcl}(\text{Ftraces}(s_2))$$

Proposition 5 implies that relation $\leq_{if}$ is reflexive ($s \leq_{if} s$) and transitive ($s_1 \leq_{if} s_2 \wedge s_2 \leq_{if} s_3 \implies s_1 \leq_{if} s_3$). Formally, it is thus a preorder, making it suitable for stepwise refinement.

## 3 Alternating Interface Automata

Real software systems are always in a single state, but the precise state of a system cannot always be derived from an observed trace. Due to non-determinism, a trace may lead to multiple states. In IAs, this is modelled as a set of states, such as the set of initial states, the set $T(q, \ell)$ for state $q$ and action $\ell$, and the set $s$ after $\sigma$ for IA $s$ and trace $\sigma$. The domain of such non-deterministic views on an IA with states $Q$ is thus the powerset of states, $\mathcal{P}(Q)$. In set of states $Q$, traces from any individual state in $Q$ may be observed.

### 3.1 Alternation

Alternation generalizes this view on automata: a system may not only be non-deterministically in multiple states, but also conjunctively. When conjunctively in multiple states, only traces which are in *all* these states may be observed. Alternation is formalized by exchanging the domain $\mathcal{P}(Q)$ for the domain $\mathcal{D}(Q)$. Formally, $\mathcal{D}(Q)$ is the *free distributive lattice*, which exist for any set $Q$ [14].

**Definition 6.** *For any set $Q$, $\mathcal{D}(Q)$ denotes the* free distributive lattice generated by *$Q$. That is, $\mathcal{D}(Q)$ is the domain of equivalence classes of terms, inductively defined by the the grammar*

$$e \quad = \quad \top \mid \bot \mid \langle q \rangle \mid e_1 \vee e_2 \mid e_1 \wedge e_2 \qquad \textit{with } q \in Q,$$

*where equivalence of terms is completely defined by the following axioms:*

$$e_1 \vee e_2 = e_2 \vee e_1 \qquad e_1 \wedge e_2 = e_2 \wedge e_1 \qquad \textit{[Commutativity]}$$
$$e_1 \vee (e_2 \vee e_3) = (e_1 \vee e_2) \vee e_3 \qquad e_1 \wedge (e_2 \wedge e_3) = (e_1 \wedge e_2) \wedge e_3 \qquad \textit{[Associativity]}$$
$$e_1 \vee (e_1 \wedge e_2) = e_1 \qquad e_1 \wedge (e_1 \vee e_2) = e_1 \qquad \textit{[Absorption]}$$
$$e \vee e = e \qquad e \wedge e = e \qquad \textit{[Idempotence]}$$
$$e_1 \vee (e_2 \wedge e_3) = (e_1 \vee e_2) \wedge (e_1 \vee e_3) \qquad e_1 \wedge (e_2 \vee e_3) = (e_1 \wedge e_2) \vee (e_1 \wedge e_3) \qquad \textit{[Distributivity]}$$
$$e \vee \top = \top \qquad e \wedge \bot = \bot \qquad \textit{[Identity]}$$

*In short, ($\mathcal{D}(Q)$, $\vee$, $\wedge$, $\bot$, $\top$) forms a distributive lattice. Expression $\langle q \rangle$ is named the embedding of $q$ in $\mathcal{D}(Q)$, and operators $\vee$ and $\wedge$ are named disjunction and conjunction, respectively. For the remainder of this paper, we make no distinction between expressions and their equivalence classes.*

*For finite $n$, we introduce the shorthand $n$-ary operators $\bigvee$ and $\bigwedge$, as follows:*

$$\bigvee\{e_1, e_2, \ldots e_n\} = e_1 \vee e_2 \vee \ldots e_n \qquad \bigvee \emptyset = \bot$$
$$\bigwedge\{e_1, e_2, \ldots e_n\} = e_1 \wedge e_2 \wedge \ldots e_n \qquad \bigwedge \emptyset = \top$$

We distinguish the embedding $\langle q \rangle \in \mathcal{D}(Q)$ from $q$ itself. We require this distinction only in Definition 18, where we will point this out. Otherwise, we do not need this distinction, so we write $q$ instead of $\langle q \rangle$.

Intuitively, disjunction $q_1 \vee q_2$ replaces the non-deterministic set $\{q_1, q_2\}$. This is formalized by extending IAs with alternation.

**Definition 7.** *An* alternating interface automaton *(AIA) is defined as a 5-tuple $(Q, I, O, T, e^0)$ where*

- *$Q$ is a set of states, and elements of $\mathcal{D}(Q)$ are referred to as* configurations*,*
- *$I$ and $O$ are disjoint sets of input and output actions, respectively,*
- *$T : Q \times (I \cup O) \to \mathcal{D}(Q)$ is a transition function, with $T(q, a) \neq \bot$ for all $a \in I$, and*
- *$e^0 \in \mathcal{D}(Q)$ is the initial configuration.*

*The domain of AIAs is denoted by $\mathcal{AIA}$. Notations for IAs are reused for AIAs, if this causes no ambiguity. For $\ell \in L$, we define $T_\ell : Q \to \mathcal{D}(Q)$ by $T_\ell(q) = T(q, \ell)$.*

Configurations $\top$ and $\bot$ are analogous to the empty set of states in an IA $s$: if $T_s(q, \ell) = \emptyset$, this means that state $q$ does not have a transition for $\ell$. In terms of input-failure refinement, not having a transition for an input means that the input is underspecified, whereas not having a transition for an output means that the output is forbidden. This distinction is made explicit in AIA by using $\top$ to represent underspecification and $\bot$ to represent forbidden behaviour. We will formalize this in Section 3.2. Definition 7 also allows output transitions to $\top$, meaning that the behaviour is unspecified after that output. Automata models which do not allow distinct configurations $\top$ and $\bot$ commonly represent such underspecified behaviour with an explicit chaotic state [3,4] instead.

We graphically represent AIAs in a similar way as IAs, with some additional rules. A transition $T(q^0, \ell) = \langle q^1 \rangle$ is represented by a single arrow from $q^0$ to $q^1$. We represent $T(q^0, \ell) = q^1 \vee q^2$ by two arrows $q^0 \xrightarrow{\ell} q^1$ and $q^0 \xrightarrow{\ell} q^2$, analogous to non-determinism in IAs. Conjunction $T(q^0, \ell) = q^1 \wedge q^2$ is shown by adding an arc between the arrows. Nested expressions are represented by successive splits, as shown in Example 8. A state $q$ without outgoing arrow for an output $\ell \in O$ represents $T(q, \ell) = \bot$, and a state without input transitions for input $\ell$ indicates $T(q, \ell) = \top$. For $\ell \in O$, a transitions $T(q, \ell) = \top$ is shown with an arrow to $\top$, denoting underspecification, but note that $\top$ is a configuration, not a state.

*Example 8.* Figure 3 shows AIA $s_A$, with $Q_A = \{q_A^0, q_A^1, q_A^2\}$, $I = \{?\mathrm{a}, ?\mathrm{b}\}$, $O = \{!\mathrm{x}, !\mathrm{y}\}$, $e_A^0 = q_A^0$ and $T$ given by the following table:

| state \ action | ?a | ?b | !x | !y |
|---|---|---|---|---|
| $q_A^0$ | $q_A^0 \wedge (q_A^1 \vee q_A^2)$ | $\top$ | $q_A^0$ | $q_A^0$ |
| $q_A^1$ | $\top$ | $\top$ | $\top$ | $\bot$ |
| $q_A^2$ | $\top$ | $q_A^0$ | $\bot$ | $q_A^2$ |

Moreover, AIA $s_B$ combines the partial specifications from Section 1.

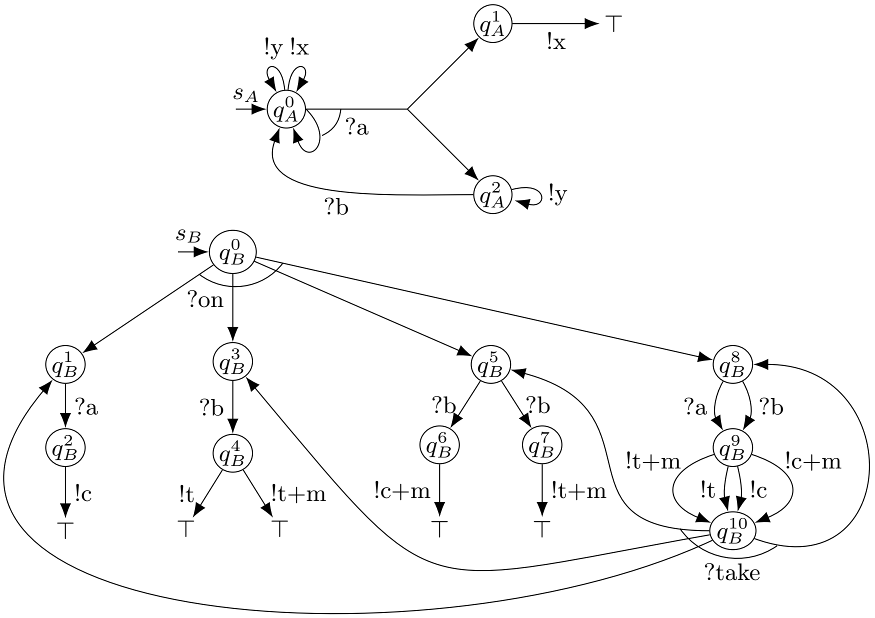

**Fig. 3.** Example AIAs $s_A$ and $s_B$.

Before defining trace semantics for AIAs, we extend the transition function from single actions to sequences of actions, by defining an after-function on AIAs. This function transforms configurations by substituting every state according to the transition function, similarly to the approach for alternating automata in [6].

**Definition 9.** *Let* $f : Q \to \mathcal{D}(Q)$ *and* $e \in \mathcal{D}(Q)$*. Then* substitution $e[f]$ *is equal to* $e$ *with all atomic propositions replaced by* $f(e)$*. Formally,* $[f] : \mathcal{D}(Q) \to \mathcal{D}(Q)$ *is a postfix operator defined by*

$$(e_1 \vee e_2)[f] = e_1[f] \vee e_2[f] \quad (e_1 \wedge e_2)[f] = e_1[f] \wedge e_2[f]$$
$$\top[f] = \top \quad \bot[f] = \bot \quad \langle q \rangle[f] = f(q)$$

**Definition 10.** *Let* $s \in \mathcal{AIA}$*. We define* after: $\mathcal{D}(Q_s) \times L^* \to \mathcal{D}(Q_s)$ *as*

$$e \text{ after}_s \ \epsilon = e \qquad e \text{ after}_s \ (\ell \cdot \sigma) = e[T_\ell] \ \text{after}_s \ \sigma$$

*Like before, we omit the subscript if clear from the context. We also define* $(s \text{ after } \sigma) = e_s^0 \text{ after}_s \ \sigma$*.*

*Example 11.* Consider $s_B$ in Figure 3. We evaluate $s_B$ after ?on ?b !t, as follows:

$$\begin{aligned} &q_B^0 \text{ after ?on ?b !t} = q_B^0[T_{\text{?on}}] \text{ after ?b !t} = T(q_B^0, \text{?on}) \text{ after ?b !t} \\ &= (q_B^1 \wedge q_B^3 \wedge q_B^5 \wedge q_B^8) \text{ after ?b !t} = (q_B^1 \wedge q_B^3 \wedge q_B^5 \wedge q_B^8)[T_{\text{?b}}] \text{ after !t} \\ &= (\top \wedge q_B^4 \wedge (q_B^6 \vee q_B^7) \wedge q_B^9) \text{ after !t} = (q_B^4 \wedge (q_B^6 \vee q_B^7) \wedge q_B^9)[T_{\text{!t}}] \\ &= (\top \wedge (\bot \vee \bot) \wedge q_B^{10}) = \bot \end{aligned}$$

Intuitively, this means that giving a tea without milk after ?on ?b is forbidden. In contrast, tea with milk is allowed, and leads to configuration $q_B^{10}$:

$$q_B^0 \text{ after ?on ?b !t+m} = (q_B^4 \wedge (q_B^6 \vee q_B^7) \wedge q_B^9)[T_{\text{!t+m}}] = \top \wedge (\bot \vee \top) \wedge q_B^{10} = q_B^{10}$$

### 3.2 Input-Failure Semantics for AIAs

IAs are equipped with input-failure semantics, based on the traces and underspecified inputs of the IA. We lift this to AIAs via the after-function, using that $\bot$ indicates forbidden behaviour, and $\top$ indicates underspecified behaviour.

**Definition 12.** *Let* $s, s' \in \mathcal{AIA}$*, and* $e \in \mathcal{D}(Q_s)$*. Then we define*

$$\begin{aligned} \text{Ftraces}_s(e) &= \{\sigma \in L^* \mid (e \text{ after}_s \ \sigma) \neq \bot\} \cup \{\sigma\overline{a} \in L^* \cdot \overline{I} \mid (e \text{ after}_s \ \sigma a) = \top\} \\ \text{Ftraces}(s) &= \text{Ftraces}_s(e_s^0) \\ s \leq_{\mathit{if}} s' &\iff \text{Ftraces}(s) \subseteq \text{Ftraces}(s') \\ s \equiv_{\mathit{if}} s' &\iff \text{Ftraces}(s) = \text{Ftraces}(s') \end{aligned}$$

Compare Definition 4 and Definition 12 for input-failure refinement for IAs and for AIAs. For AIAs, refinement is defined directly over their Ftraces, whereas for IA, the input-failure closure of the Ftraces is used for the right-hand model (and optionally for the left-hand model, according to Proposition 5). In this regard, AIAs are a more direct and natural representation of input-failure traces, since the input-failure closure is not needed.

**Proposition 13.** *For* $s \in \mathcal{AIA}$*,* Ftraces$(s)$ *is input-failure closed.*

Another motivation to represent input-failure traces with AIAs is the connection between the distributive lattice $\mathcal{D}(Q)$ and the lattice of sets of input-failure traces: $\wedge$ and $\vee$ are connected to intersection and union of input-failure traces, respectively, and $\top$ and $\bot$ represent the largest and smallest possible input-failure trace sets.

**Proposition 14.** *Let $s \in \mathcal{AIA}$, and $e, e' \in \mathcal{D}(Q_s)$. Then*

1. $\text{Ftraces}(e \wedge e') = \text{Ftraces}(e) \cap \text{Ftraces}(e')$
2. $\text{Ftraces}(e \vee e') = \text{Ftraces}(e) \cup \text{Ftraces}(e')$
3. $\text{Ftraces}(\bot) = \emptyset$
4. $\text{Ftraces}(\top) = \mathcal{FT}_{I,O}$
5. $\text{Ftraces}(e) = \{\epsilon\} \cup \{\overline{a} \in \overline{I_s} \mid e \text{ after } a = \top\} \cup (\bigcup_{\ell \in L_s} \ell \cdot \text{Ftraces}(e \text{ after } \ell)) \qquad \textit{if } e \neq \bot$

Propositions 14.3 and 14.5 show why Definition 7 does not allow transitions to $T(q, a) = \bot$ for an input $a$: in that case, $\text{Ftraces}(q)$ would contain trace $\epsilon$, but it would not contain extension $a$ nor $\overline{a}$ of $\epsilon$, meaning that after trace $\epsilon$ it is not allowed to accept nor to refuse $a$.

We can lift configurations $\top$ and $\bot$, as well as $\wedge$ and $\vee$, to the level of AIAs. This provides the building blocks to compose specifications. Specifications $s_\top$ and $s_\bot$ can be used to specify that any or no behaviour is considered correct, respectively. The operators $\wedge$ and $\vee$ on specifications fulfill the same role as existing operators in substitutivity refinement [7], and have similar properties, described in Proposition 14.

**Definition 15.** *Let $s_1, s_2 \in \mathcal{AIA}$. Without loss of generality*[1]*, assume that $Q_1$ and $Q_2$ are disjoint. We define*

$$s_\top = (\emptyset, I, O, \emptyset, \top) \qquad s_1 \wedge s_2 = (Q_1 \cup Q_2, I, O, T_1 \cup T_2, e_1^0 \wedge e_2^0)$$
$$s_\bot = (\emptyset, I, O, \emptyset, \bot) \qquad s_1 \vee s_2 = (Q_1 \cup Q_2, I, O, T_1 \cup T_2, e_1^0 \vee e_2^0)$$

**Proposition 16.** *Let $i, i', s, s' \in \mathcal{AIA}$. Then*

$$\begin{aligned} i \leq_{if} s \textit{ and } i \leq_{if} s' &\iff i \leq_{if} (s \wedge s') \\ i \leq_{if} s \textit{ or } i \leq_{if} s' &\implies i \leq_{if} (s \vee s') \\ i \leq_{if} s \textit{ and } i' \leq_{if} s &\iff (i \vee i') \leq_{if} s \\ i \leq_{if} s \textit{ or } i' \leq_{if} s &\implies (i \wedge i') \leq_{if} s \\ &i \leq_{if} s_\top \\ &i \not\leq_{if} s_\bot \qquad \textit{if } e_i^0 \neq \bot \end{aligned}$$

The converse of statement (2) does not hold. As a counter-example, choose $\text{Ftraces}(i) = \{\epsilon, x, y\}$, $\text{Ftraces}(s_1) = \{\epsilon, x\}$ and $\text{Ftraces}(s_2) = \{\epsilon, y\}$. In that case, $i \leq_{if} s_1 \vee s_2$ holds, but $i \not\leq_{if} s_1$ and $i \not\leq_{if} s_2$. The converse of statement (4) can be disproven similarly.

[1] If $Q_1$ and $Q_2$ are not disjoint, the disjoint union $Q_1 \uplus Q_2$ can be used instead of $Q_1 \cup Q_2$. The transition functions of $s_1 \wedge s_2$ and $s_1 \vee s_2$ should be adjusted accordingly.

### 3.3 AIA Determinization

In case of nestings of $\wedge$ and $\vee$, the after-set $s$ after $\sigma$ may not be clear immediately, so a transition function producing configurations without $\wedge$ and $\vee$ is easier to interpret. For this reason, we lift the notions of determinism and determinization from IAs [11] to the alternating setting.

**Definition 17.** *Let $s \in \mathcal{AIA}$ and $e \in \mathcal{D}(Q_s)$. Then $e$ is* deterministic *if $e = \top$ or $e = \bot$ or $e = \langle q \rangle$ for some $q \in Q_s$. Furthermore, $s$ is deterministic if for all $\sigma \in L^*$, configuration $s$* after *$\sigma$ is deterministic.*

Compare the notions of determinism for IAs and AIAs. For every trace $\sigma$, a deterministic IA $s$ is in a singleton state ($s$ after $\sigma$) $= \{q\}$, unless ($s$ after $\sigma$) $= \emptyset$ (that is, $\sigma$ is not a trace of $s$). For AIAs, this singleton set $\{q\}$ is replaced by the embedding $\langle q \rangle$, and $\emptyset$ is replaced by $\top$ or $\bot$, depending on whether this set was reached by an undespecified action or a forbidden action.

We now define determinization, where we require the distinction between $\langle q \rangle$ and $q$ to avoid ambiguity.

**Definition 18.** *Let $s \in \mathcal{AIA}$. We define* $\det : \mathcal{D}(Q_s) \to \mathcal{D}(\mathcal{D}(Q_s) \setminus \{\top, \bot\})$ *as*

$$\det(e) = \begin{cases} \top & \textit{if } e = \top \\ \bot & \textit{if } e = \bot \\ \langle e \rangle & \textit{otherwise} \end{cases}$$

*The* determinization of *$s$, or* $\det(s) \in \mathcal{AIA}$, *is defined as*

$$\det(s) = (\mathcal{D}(Q_s) \setminus \{\top, \bot\}, I, O, T_{\det(s)}, \det(e_s^0)), \textit{ with}$$
$$T_{\det(s)}(e, \ell) = \det(e \text{ after}_s\ \ell) \qquad \textit{for } \ell \in L$$

**Proposition 19.** *For $s \in \mathcal{AIA}$,* $\det(s)$ *is deterministic.*

*Example 20.* Figure 4 shows (the reachable part of) the determinizations of $s_A$ and $s_B$ from Figure 3. In $\det(s_A)$, state $q_A^0 \wedge q_A^2$ has no outgoing !x-transition. This expresses $T_{\det(s_A)}(q_A^0 \wedge q_A^2, !\text{x}) = \bot$, which is because $q_A^2$ has no $x$-transition, so $T_A(q_A^0, !\text{x}) = \bot$. In contrast, state $q_A^0 \wedge q_A^2$ has an outgoing ?a-transition, $T_{\det(s_A)}(q_A^0 \wedge q_A^2, ?\text{a}) \neq \top$, because $q_A^0$ has an ?a-transition, $T_A(q_A^0, ?\text{a}) \neq \top$.

Example 20 shows that an input is specified by a conjunction of states in the determinization if *any* of the individual state specify this input, whereas an output is allowed by a conjunction of states only if *all* of the individual state allow this output. In the setting of IA, [11] already established that this works in a reversed way for non-determinism, following their definition of determinization: *all* individual states of a disjunction should specify an input to specify it in the determinization, and *any* individual state should allow an output to allow it in the determinization. Their so-called *input-universal determinization* is an instance of the determinization from Definition 18, using only disjunctions.

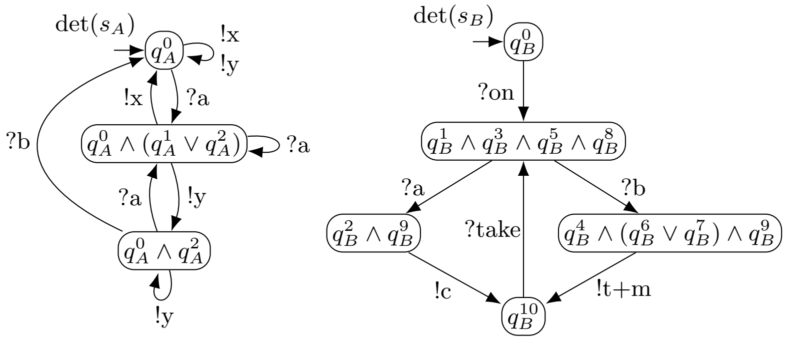

**Fig. 4.** Examples of determinization.

This duality arises from Definition 10 of after, since the determinization directly represents the after-function: the determinizations in Example 20 correspond to the after-sets such as those derived in Example 11. This correspondence is formalized in Proposition 21.

**Proposition 21.** *Let* $s \in \mathcal{AIA}$ *and* $\sigma \in L^*$. *Then*

$$(\text{det}(s) \text{ after } \sigma) = \text{det}(s \text{ after } \sigma).$$

**Proposition 22.** *Let* $s \in \mathcal{AIA}$. *Then* $\text{Ftraces}(s) = \text{Ftraces}(\text{det}(s))$.

**Corollary 23.** *Let* $s \in \mathcal{AIA}$. *Then* $s \equiv_{if} \text{det}(s)$.

A known result [6] is that alternating automata are exponentially more succinct than non-deterministic automata, and double exponentially more succinct than deterministic automata. Although alternating automata are not a special case of AIAs (as AIAs lack the accepting and non-accepting states of alternating automata), we expect AIAs to be exponentially more succinct than IAs, as well.

### 3.4 Connections between IAs and AIAs

IAs and AIAs are used to represent sets of input-failure traces, and are in that sense interchangeable. First, we show that any IA can be translated to an AIA.

**Definition 24.** *For* $s \in \mathcal{IA}$, *the* AIA induced by $s$ *is defined as* $\text{AIA}(s) = (Q_s, I_s, O_s, T, \bigvee Q_s^0) \in \mathcal{AIA}$, *where for all* $q \in Q_s$ *and* $\ell \in L$:

$$T(q, \ell) = \begin{cases} \top & \text{if } \ell \in I \text{ and } q \xrightarrow{\ell}\!\!\!\!/ \\ \bigvee T_s(q, \ell) & \text{otherwise} \end{cases}$$

**Proposition 25.** *Let* $s \in \mathcal{IA}$. *Then* $\text{Ftraces}(\text{AIA}(s)) = \text{fcl}(\text{Ftraces}(s))$.

**Corollary 26.** *Let* $s_1, s_2 \in \mathcal{IA}$. *Then* $s_1 \leq_{if} s_2 \iff \text{AIA}(s_1) \leq_{if} \text{AIA}(s_2)$

Definition 29 formalizes how disjunction in an AIA corresponds to non-determinism in IA. Specifically, if no transitions are present for some output in an IA, then the transition function of the corresponding AIA gives $\bigvee \emptyset = \bot$ for this output, analogous to the explicit case $\top$ for inputs. Note that the graphical representation of an IA and that of its induced AIA are the same.

The translation from AIAs to IAs is more involved. For disjunctions of states $(q \text{ after } \ell) = q_1 \vee q_2$, the translation of Definition 24 can simply be inverted, but this is not possible for conjunctions. As such, we represent any configuration by its unique disjunctive normal form.

**Definition 27.** *Let* $e \in \mathcal{D}(Q)$*. Then* $\mathrm{DNF}(e)$ *is the smallest set in* $\mathcal{P}(\mathcal{P}(Q))$ *such that* $e = \bigvee\{\bigwedge Q' \mid Q' \in \mathrm{DNF}(e)\}$.

The set $\mathrm{DNF}(e)$ can be constructed by using the axioms from Definition 6.

*Example 28.* To find $\mathrm{DNF}(q^1 \vee (q^2 \wedge (q^1 \vee q^3)))$, we first rewrite the expression by using distributivity, associativity, commutativity and absorbtion, as follows:

$$q^1 \vee (q^2 \wedge (q^1 \vee q^3)) = q^1 \vee (q^2 \wedge q^1) \vee (q^2 \wedge q^3) = q^1 \vee (q^2 \wedge q^3)$$

So we find $\mathrm{DNF}(q^1 \vee (q^2 \wedge (q^1 \vee q^3))) = \{\{q^1\}, \{q^2, q^3\}\}$. Two other examples are $\mathrm{DNF}(\bot) = \mathrm{DNF}(\bigvee \emptyset) = \emptyset$ and $\mathrm{DNF}(\top) = \mathrm{DNF}(\bigvee\{\bigwedge \emptyset\}) = \{\emptyset\}$.

**Definition 29.** *Let* $s \in \mathcal{AIA}$*. Then the* induced IA *of* $s$ *is defined as*

$$\mathrm{IA}(s) = (\mathcal{P}(Q_s), I, O, T, \mathrm{DNF}(e_s^0)) \in \mathcal{IA}, \textit{ with for } Q \subseteq Q_s \textit{ and } \ell \in L\text{:}$$

$$T(Q, \ell) = \begin{cases} \mathrm{DNF}((\bigwedge Q)[T_{s\ell}]) \setminus \{\emptyset\} & \textit{if } \ell \in I \\ \mathrm{DNF}((\bigwedge Q)[T_{s\ell}]) & \textit{if } \ell \in O \end{cases}$$

A state of $\mathrm{IA}(s)$ acts as the conjunction of the corresponding states in $s$. In particular, a singleton state $\{q\}$ in $\mathrm{IA}(s)$ acts as the contained state $q$ in $s$, and state $\emptyset$ in $\mathrm{IA}(s)$ acts as a chaotic state, having $\mathrm{Ftraces}_{\mathrm{IA}(s)}(\emptyset) = \mathcal{FT}_{I,O}$.

**Proposition 30.** *Let* $s \in \mathcal{AIA}$*. Then* $\mathrm{Ftraces}(s) = \mathrm{fcl}(\mathrm{Ftraces}(\mathrm{IA}(s)))$.

**Corollary 31.** *Let* $s_1, s_2 \in \mathcal{AIA}$*. Then* $s_1 \leq_{\mathit{if}} s_2 \iff \mathrm{IA}(s_1) \leq_{\mathit{if}} \mathrm{IA}(s_2)$

## 4 Testing Input-Failure Refinement

So far, we have introduced refinement as a way of specifying correctness of one model with respect to another. Often, a specification is indeed a model, but we use it to ensure correctness of a real-world software implementation. To this end, we assume that this implementation behaves like a IA. We cannot see the actual states and transitions of this IA, but we can provide inputs to it and observe its outputs. We assume that this IA must have an initial state, i.e. it is *non-empty*.

**Definition 32.** *[1] An IA* $i$ *is* empty *if* $Q_i^0 = \emptyset$.

In this section, we introduce a basis for *model-based testing* with AIAs, analogously to ioco test case generation [15]. Given a specification AIA, we derive a testing experiment on non-empty implementation IAs, in order to observe whether input-failure refinement holds with respect to the specification. This requires an extension of input-failure refinement to these domains.

**Definition 33.** *Let $i \in \mathcal{IA}$ and $s \in \mathcal{AIA}$. Then*

$$i \leq_{if} s \iff \text{Ftraces}(i) \subseteq \text{Ftraces}(s).$$

### 4.1 Testers for AIA Specifications

From a given specification AIA, we derive a tester. We model this tester as an IA as well, which can communicate with an implementation IA through a form of parallel composition. The tester eventually concludes a verdict, indicating whether the observed behaviour is allowed. To communicate, the inputs of the implementation must be outputs for the tester, and vice versa (note that $I$ and $O$ denote the inputs and outputs for the *implementation*, respectively). The tester should not block or ignore outputs from the implementation, meaning that the tester should be input-enabled. If the tester intends to supply an input to the implementation, it should also be prepared for a refusal of that input. A verdict is given by means of special states **pass** or **fail**. Lastly, to give consistent verdicts, a tester should be deterministic. This leads to the following definition of testers.

**Definition 34.** *A* tester for (an IA or AIA with) inputs $I$ and outputs $O$ *is a deterministic, input-enabled IA* $t = (Q_t,\ O,\ I \cup \overline{I},\ T,\ q_t^0)$ *with* $\mathbf{pass}, \mathbf{fail} \in Q_t$*, such that* **pass** *and* **fail** *are sink-states with* $\text{out}(\mathbf{pass}) = \text{out}(\mathbf{fail}) = \emptyset$*, and* $a \in \text{out}(q) \iff \overline{a} \in \text{out}(q)$ *for all* $q \in Q_t$ *and* $a \in I$.

Testing is performed by a special form of parallel composition of a tester and an implementation. If the tester chooses to perform an input while the implementation also chooses to produce an output, this results in a race condition. In such a case, both the input or the output can occur during test execution. We assume a synchronous setting, in which the implementation and specification agree on the order in which observed actions are performed (in contrast to e.g. a queue-based setting [13], in which all possible orders are accounted for). These assumptions are in line with the assumptions in e.g. ioco-theory [15], and lead to the following definition of test execution.

**Definition 35.** *Let $i \in \mathcal{IA}$ be non-empty, and let $t$ be a tester for $i$. We write* $q_t \rceil\!| \, q_i$ *for* $(q_t, q_i) \in Q_t \times Q_i$. *Then* test execution of $i$ against $t$*, denoted* $t \rceil\!| \, i$*, is defined as* $(Q_t \times Q_i,\ \emptyset,\ I \cup \overline{I} \cup O,\ T,\ q_t^0 \rceil\!| \, q_i^0) \in \mathcal{IA}$*, with*

$$T(q_t \rceil\!| \, q_i, \ell) = \{q_t' \rceil\!| \, q_i' \mid q_t \xrightarrow{\ell} q_t',\ q_i \xrightarrow{\ell} q_i'\} \qquad \textit{for } \ell \in L$$

$$T(q_t \rceil\!| \, q_i,\ \overline{a}) = \{q_t' \rceil\!| \, q_i \mid q_t \xrightarrow{\overline{a}} q_t',\ q_i \not\xrightarrow{a}\} \qquad \textit{for } a \in I$$

*We say that* $i$ **fails** $t$ *if* $q_t^0 \rceil\!| \, q_i^0 \xrightarrow{\sigma} \mathbf{fail} \rceil\!| \, q_i$ *for some* $\sigma$ *and* $q_i$*, and* $i$ **passes** $t$ *otherwise.*

We reuse the notions of *soundness* and *exhaustiveness* from [15], to express whether a tester properly tests for a given specification.

**Definition 36.** *Let $s \in \mathcal{AIA}$ and let $t$ be a tester for $s$. Then $t$ is* sound *for $s$ if for all $i \in \mathcal{IA}$ with inputs $I$ and outputs $O$, $i$* **fails** *$t$ implies $i \not\leq_{if} s$. Moreover, $t$ is* exhaustive *for $s$ if for all $i \in \mathcal{IA}$, $i$* **passes** *$t$ implies $i \leq_{if} s$.*

A simple attempt to translate specification AIA $s$ to a sound and exhaustive tester would be similar to the determinization of $s$, but replacing every occurence of $\bot$ and $\top$ by **fail** and **pass**, respectively.

$$f_t(e) = \begin{cases} \textbf{fail} & \text{if } e = \bot \\ \textbf{pass} & \text{if } e = \top \\ e & \text{otherwise} \end{cases}$$

Taking special care of input failures, the function $f_t$ then induces a tester $(\mathcal{D}(Q_s) \cup \{\textbf{pass}, \textbf{fail}\}, O, I \cup \overline{I}, T, f_t(e_s^0))$, with

$$T(e, \ell) = \{f_t(e \text{ after}_s\ \ell)\} \qquad \text{for } e \in \mathcal{D}(Q_s), \ell \in L$$

$$T(v, \ell) = \begin{cases} \{v\} & \text{if } \ell \in O \\ \emptyset & \text{if } \ell \in I \end{cases} \qquad \text{for } v \in \{\textbf{pass}, \textbf{fail}\}$$

$$T(e, \overline{a}) = \begin{cases} \{\textbf{pass}\} & \text{if } (e \text{ after}_s\ a) = \top \\ \{\textbf{fail}\} & \text{otherwise} \end{cases} \qquad \text{for } e \in \mathcal{D}(Q_s), a \in I$$

This tester is sound and complete for $s$: each possible input-failure trace is in Ftraces($s$) if and only if it does not lead to **fail**, by construction. Here, we make use of the fact that Ftraces($\bot$) $= \emptyset$, meaning that $\bot$ cannot be implemented correctly by a non-empty IA and can thus be replaced by **fail**. Likewise, Ftraces($\top$) $= \mathcal{FT}_{I,O}$ means that $\top$ is always implemented correctly, and can be replaced by **pass**.

However, this tester is quite inefficient. If a tester reaches **pass** after both $\sigma a$ and $\sigma\overline{a}$, then this input $a$ does not need to be tested after $\sigma$. Specifically, this is the case if and only if trace $\sigma a$ leads to specification configuration $\top$. We thus improve the tester for a given specifications as follows.

**Definition 37.** *Let $s \in \mathcal{AIA}$. Then* tester($s$) $\in \mathcal{IA}$ *is defined as*

$$\text{tester}(s) = (\mathcal{D}(Q_s) \cup \{\textbf{pass}, \textbf{fail}\}, O, I \cup \overline{I}, T, f_t(e_s^0)), \text{ with } f_t \text{ as before, and}$$

$$T(e, \ell) = \begin{cases} \{f_t(e \text{ after}_s\ \ell)\} & \text{if } \ell \in O, \text{ or } \ell \in I \text{ and } (e \text{ after}_s\ \ell) \neq \top \\ \emptyset & \text{if } \ell \in I \text{ and } (e \text{ after}_s\ \ell) = \top \end{cases} \qquad \text{for } \ell \in L$$

$$T(e, \overline{a}) = \begin{cases} \emptyset & \text{if } (e \text{ after}_s\ a) = \top \\ \{\textbf{fail}\} & \text{otherwise} \end{cases} \qquad \text{for } e \in \mathcal{D}(Q_s), a \in I$$

$$T(v, \ell) = \begin{cases} \{v\} & \text{if } \ell \in O \\ \emptyset & \text{if } \ell \in I \end{cases} \qquad \text{for } v \in \{\textbf{pass}, \textbf{fail}\}, \ell \in L$$

*Example 38.* The tester for $s_B$ in Figure 3 is shown in Figure 5.

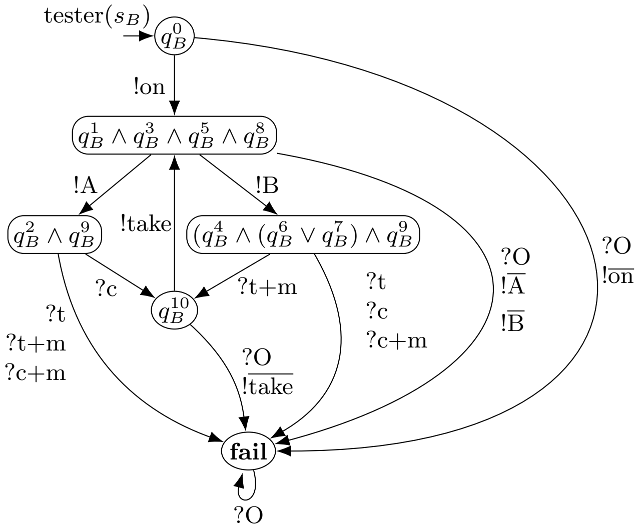

**Fig. 5.** The tester for the vending machine. The label ?O denotes a transition for every label in O. Remark that inputs for $s_B$ are outputs for tester($s_B$), and vice versa.

Theorem 39 shows that soundness and exhaustiveness of a tester corresponds to refinement of the corresponding AIA.

**Theorem 39.** *Let* $s_1, s_2 \in \mathcal{AIA}$. *Then*

*1* tester($s_1$) *is sound and exhaustive for* IA($s_1$)

*2* tester($s_1$) *is sound for* $s_2 \iff s_2 \leq_{if} s_1$

*3* tester($s_1$) *is exhaustive for* $s_2 \iff s_1 \leq_{if} s_2$

### 4.2 Test Cases for AIA Specifications

In [15], an algorithm was introduced to generate *test cases*. These are testers as in Definition 34 with additional restrictions, so that they can be used as unambiguous instructions to test a system. In particular, states of a test case should have at most one outgoing input transition. This ensures that no choice between different inputs has to be resolved during test execution. Additionaly, all paths of a test case lead to **pass** or **fail** in a finite number of steps, to ensure that test execution terminates with a verdict.

**Definition 40.** *A tester t for I and O is a* test case *if*

– *for all* $q_t \in Q_t$, $|\text{out}(q_t)| \leq 1$, *and*
– *there are no infinite sequences* $q_t^0, q_t^1, \ldots$ *for* $q_t^0, q_t^1, \ldots \in Q_t \setminus \{\textbf{pass}, \textbf{fail}\}$ *such that* $q_t^0 \xrightarrow{\ell^0} q_t^1 \xrightarrow{\ell^1} \ldots$

The test case generation algorithm of [15] is non-deterministic, since it must choose at most one inputs in every state, and it must choose when to stop testing. We avoid defining a separate test case generation algorithm, and instead use Theorem 39 to obtain sound test cases. If specification $s_1$ is weakened to $s_2$, such that tester$(s_2)$ is a test case, then soundness of tester$(s_2)$ for $s_1$ is guaranteed by the theorem. Such a weakened *singular specification* $s_2$ describes a finite, tree-shaped part of the original specification $s_1$.

**Definition 41.** *Let* $s_1, s_2 \in \mathcal{AIA}$. *Then* $s_2$ *is a* singular specification for $s_1$ *if* $Q_2$ *is a finite subset of* $L^*$, *with* $e_2^0 \in \{\epsilon, \top, \bot\}$, $e_1^0 = \top \implies e_2^0 = \top$ *and* $e_2^0 = \bot \implies e_1^0 = \bot$, *and having that for every* $\sigma \in Q_2$, *the following holds:*

1. $T_2(\sigma, \ell) = \bot \implies (s_1 \text{ after } \sigma\ell) = \bot$ *for* $\ell \in L$,
2. $(s_1 \text{ after } \sigma\ell) = \top \implies T_2(\sigma, \ell) = \top$ *for* $\ell \in L$
3. $T_2(\sigma, \ell)$ *is either* $\bot$ *or* $\top$ *or* $\sigma\ell$ *for* $\ell \in L$, *and*
4. *there is at most one* $a \in I$ *with* $T(\sigma, a) \neq \top$.

It can be created from $s_1$ similarly to test case generation in [15]. In every state $\sigma$ of the tree $s_1$, we either decide to pick one input specified in $s_1$ and also specify that in $s_2$; or we do not specify any input, but only outputs; or we leave any successive behaviour unspecified ($\top$).

Test cases based on singular specifications are inherently sound, and for any incorrect implementation, it is possible to find a singular specification which induces a test case to detects this incorrectness.

**Theorem 42.** *If* $s_2$ *is a singular specification for* $s_1$, *then* tester$(s_2)$ *is a sound test case for* $s_1$.

**Theorem 43.** *Let* $i \in \mathcal{IA}$ *and* $s_1 \in \mathcal{AIA}$. *If* $i \not\leq_{\mathit{if}} s_1$, *then there is a singular specification* $s_2$ *for* $s_1$ *such that* $i$ **fails** tester$(s_2)$.

*Example 44.* Specification $s_B$ in Figure 3 can be weakened to singular specification $s_C$ shown in Figure 6. Indeed, $s_B \leq_{\mathit{if}} s_C$ holds, which can be established by comparing $s_C$ with det$(s_B)$ in Figure 4. Therefore tester$(s_C)$ is a sound test case for $s_B$.

## 5 Conclusion and Future Work

Alternating interface automata serve as a natural and direct representation for sets of input-failure traces, and therefore also for refinement of systems with inputs, outputs, non-determinism and conjunction. We have used the observational nature of input-failure traces to define testers, describing an experiment to observationally establish refinement of a black-box system.

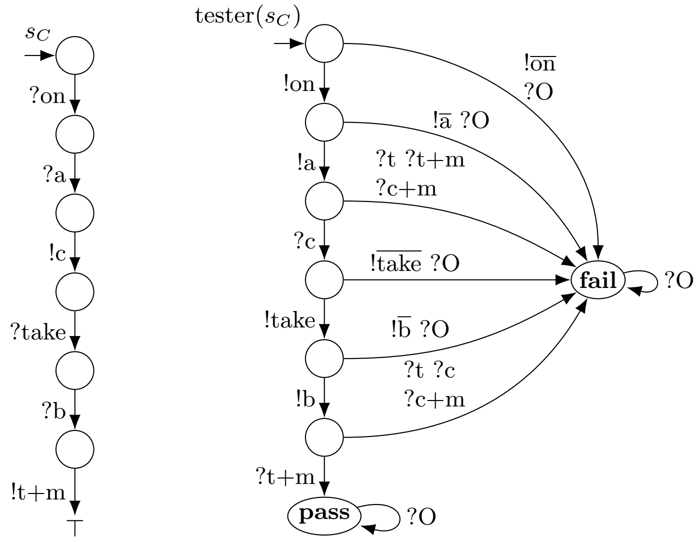

**Fig. 6.** A weakened version $s_C$ of the vending machine, and the test case tester($s_C$). Question and exclamation marks are interchanged in tester($s_C$) to indicate that the input and output alphabets have been interchanged with respect to $s_C$.

The disjunction and conjunction of alternation brings interface automata specifications closer to the realm of logic and lattice theory. On the theoretical side, a possible direction is to extend configurations from distributive lattices to a full logic. On the practical side, classical testing techniques acting on logical expressions, such as combinatorial testing, could be translated to our black-box configurations of states.

Possible criticism on our running example of a vending machine $s_B$ in Figure 3 may be that its representation as an AIA is not concise, since the determinization det($s_B$) is much smaller and more understandable than $s_B$ itself. This is because the individual specifications offer a choice between outputs, such as tea with or without milk, whereas the intersection of all choices is singleton. A more natural encoding for this example is to express the types of drink with data data parameters, and the restrictions on them by logical constraints. This requires an automaton model in style of symbolic transition systems [9], which could be enriched with the concepts of alternation of AIAs.

Interface automata typically contain internal transitions, and the interaction between internal behaviour and alternation is not immediately clear. A possible approach to extend AIAs with internal behaviour is to lift the $\epsilon$-closure of [1], the set of states reachable via internal transitions, to the level of configurations.

**Acknowledgement.**

We thank Jan Tretmans and Frits Vaandrager for their valuable feedback.
